# Influence of Two Depuration Periods on the Activity and Transcription of Antioxidant Enzymes in Tilapia Exposed to Repeated Doses of Cylindrospermopsin under Laboratory Conditions 

**DOI:** 10.3390/toxins6031062

**Published:** 2014-03-13

**Authors:** Victoria Ríos, Remedios Guzmán-Guillén, Isabel M. Moreno, Ana I. Prieto, María Puerto, Angeles Jos, Ana M. Cameán

**Affiliations:** Area of Toxicology, Faculty of Pharmacy, University of Sevilla, C/Profesor García González 2, Sevilla 41012, Spain; E-Mails: victoriarios@us.es (V.R.); rguzman1@us.es (R.G.-G.); imoreno@us.es (I.M.M.); anaprieto@us.es (A.I.P.); mariapuerto@us.es (M.P.); angelesjos@us.es (A.J.)

**Keywords:** cylindrospermopsin, depuration, glutathione transferase, glutathione peroxidise, tilapia

## Abstract

The cyanobacterial toxin Cylindrospermopsin (CYN), a potent protein synthesis inhibitor, is increasingly being found in freshwater bodies infested by cyanobacterial blooms worldwide. Moreover, it has been reported to be implicated in human intoxications and animal mortality. Recently, the alteration of the activity and gene expression of some glutathione related enzymes in tilapias (*Oreochromis niloticus*) exposed to a single dose of CYN has been reported. However, little is known about the effects induced by repeated doses of this toxin in tilapias exposed by immersion and the potential reversion of these biochemical alterations after two different depuration periods (3 or 7 days). In the present study, tilapias were exposed by immersion to repeated doses of a CYN-containing culture of *Aphanizomenon ovalisporum* during 14 days, and then were subjected to depuration periods (3 or 7 days) in clean water in order to examine the potential reversion of the effects observed. The activity and relative mRNA expression by real-time polymerase chain reaction (PCR) of the antioxidant enzymes glutathione peroxidase (GPx) and soluble glutathione-*S*-transferases (sGST), and also the sGST protein abundance by Western blot analysis were evaluated in liver and kidney of fish. Results showed significant alterations in most of the parameters evaluated and their recovery after 3 days (GPx activity, sGST relative abundance) or 7 days (GPx gene expression, sGST activity). These findings not only confirm the oxidative stress effects produced in fish by cyanobacterial cells containing CYN, but also show the effectiveness of depuration processes in mitigating the CYN-containing culture toxic effects.

## 1. Introduction

Cyanobacterial toxins have become recognized as a potential hazard in drinking water worldwide [1]. Among them, Cylindrospermopsin (CYN) is an emerging toxin [2], which can be produced by several known freshwater cyanobacteria species, such as *Cylindrospermopsis raciborskii*, *Aphanizomenon ovalisporum*, *Raphidiopsis curvata*, and *Umezakia natans* [3,4,5]. This toxin is a tricyclic alkaloid comprised of a guanidine entity along with a uracil moiety potentially responsible for the toxicity [6]. It is stable in varying heat, light and pH conditions and also highly water-soluble [7]. Thus, the concentration of CYN dissolved in water can constitute as much as 90% of total CYN available [8,9]. The environmental levels of CYN detected worldwide are dependent on the season and the area of detection and the toxin producing species. Thus, in Australia, the levels of CYN produced by natural blooms of *C. raciborskii* and *A. ovalisporum* oscillated between 0.3 µg/L to 92 µg/L and 4–120 µg/L, respectively. Higher levels were measured in aquaculture ponds and farm dams produced by *A. ovalisporum* (589–800 µg/L). In Europe, the CYN levels detected in natural waters were lower than in Australia, thus, in Germany CYN was found in maximum concentrations of 1.80 µg/L and 11.75 µg/L produced by *C. raciborskii* and *A. gracile* in different lakes. In France, CYN was detected in eleven water bodies with concentrations up to 1.95 µg/L. In Italy, the presence of CYN was observed in levels in a range from 0.41 to 42.3 µg/L and in Spain CYN was measured in a level up to 9.4 µg/L. In Florida (USA), CYN was positively quantified in a range from 8.07 to 97.12 µg/L [10]. 

Different studies have revealed the environmental toxicity of CYN towards different groups of organisms, including amphibians [11], gastropods [12], fish [13,14], plants [15], and aquatic macrophytes [16], as well as antibacterial activity [17]. CYN exposure has hepatotoxic, nephrotoxic and general cytotoxic effects and can also lead to fetal toxicity, tumor initiation, micronucleus induction and chromosome loss [4,5]. The toxicity of CYN is due to the inhibition of glutathione and protein synthesis, the inhibition of cytochrome P450, and direct interaction with DNA [18,3]. A depletion of hepatic glutathione has been proved in fish *in vivo* [19] and *in vitro* [20]. More recently, some studies have shown that pure CYN is able to induce oxidative stress in tilapia fish (*Oreochromis niloticus*) when they are exposed to a single dose of CYN by oral route [13,19] and intraperitoneal (i.p.) injection [14]. Alterations in the activity and gene expressions of glutathione-*S*-transferases (GST) and glutathione peroxidase (GPx) in response to a single dose of CYN, has been previously investigated in our laboratory in tilapia fish [13,21]. Results showed that tilapia exposed by gavage to 200 and 400 µg/kg bw of pure CYN and sacrificed after 24 h had alterations in different biomarkers including activity and gene expression of the enzymes GPx and GST [13]. Gutiérrez-Praena *et al.* [21] proved that the type of exposure and the time of sacrifice played a role in the activity and relative mRNA expression by real time PCR of GPx and GST and the sGST protein abundance.

Under natural conditions, fish are exposed to cyanobacterial blooms for sub-chronic periods, but CYN toxicological studies for long exposure periods are very scarce at the time [22], especially in the case of fish. In this sense, Guzmán-Guillén *et al.* [23] showed an involvement of oxidative stress as a mechanism of toxic action of CYN in tilapia after sub-chronic exposure to cyanobacterial cells containing CYN (10 and 100 μg CYN/L), for 7 and 14 day by immersion route, mimicking natural exposure). It was demonstrated that subchronic exposure to low concentrations of cyanobacterial cells containing CYN exceeding 10 μg/L should be considered of particularly high risk for fish, because evident histopathological changes were found from this concentration [24].

Several authors have studied the effect of different depuration periods on stress biomarker levels in fish and clams exposed to contaminants [25,26,27,28,29], revealing that depuration reduces the oxidative stress. However in the case of cyanobacterial toxins, depuration studies in aquatic organism are relatively scarce. Ozawa *et al.* [30] studied the accumulation and depuration of microcystins (MCs) in freshwater snails, and Vasconcelos *et al.* [31] and Tricarico *et al.* [32] in the crayfish *Procambarus clarkii*. Galanti *et al.* [33] investigated the detoxification dynamic of MC-LR in the shrimp *Palaemonetes argentinus.* Moreover, Kankaanpää *et al.* [34] studied the depuration mechanism of nodularin in mussel (*Mytilus edulis*). As far as we know, in the case of CYN, only Saker *et al.* [35] investigated the accumulation and depuration of CYN in the freshwater mussel *Anodonta cygnea*. 

Taking all these data into account, the aim of this study was to investigate the transcriptional and catalytic response of the glutathione (GSH) related enzymes GPx and sGST, in tilapia fish (liver and kidney) after a dose repeated exposure (at environmentally relevant concentration) of a CYN-containing culture of *Aphanizomenon ovalisporum* during 14 days, and the influence of two different depuration periods (3 or 7 days) in these parameters. After CYN-exposure and both depuration periods, mRNA levels and enzymatic activities were measured for GPx and sGST, including protein abundance of sGST. 

## 2. Results and Discussion

### 2.1. Results

No fish died and they exhibited no obvious signs of stress during the acclimation period, the exposure to *A. ovalisporum* culture or during the depuration periods.

#### 2.1.1. Gluthatione Peroxidase and Gluthatione-*S*-Transferase Activities

**Figure 1:**
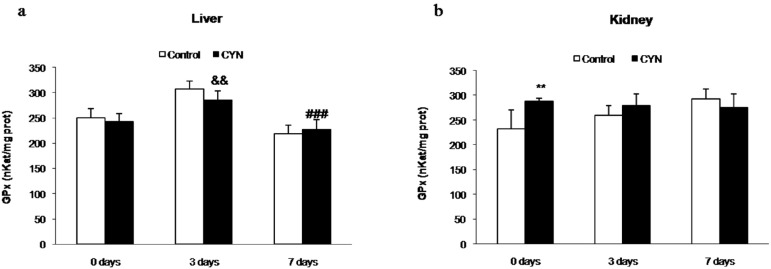
Glutathione peroxidase (GPx) activity (nkatals/mg protein) in liver (**a**) and kidney (**b**) of fish exposed by immersion to repeated doses of CYN-containing culture (equivalent to 10 µg CYN/L, added into aquaria every two days) for 14 days and later sacrificed (0 day) or depurated (3 or 7 days). The values are expressed as mean ± SE (*n* = 8). The significance levels observed are ** *p* < 0.01 in comparison with their respective control group, && *p* < 0.01 when CYN exposed and no depurated fish (0 day group) and fish depurated at different times (3 or 7 days group) are compared, and ### *p* < 0.001 when comparing CYN-depurated fish at 3 to 7 days of depuration.

In general, no changes in GPx activity were observed in the liver of fish exposed to CYN-containing culture (equivalent to 10 μg CYN/L) in any of the test groups in comparison with their controls (Figure 1a). In the kidneys, a significant increase in GPx activity was observed in fish treated with CYN and not depurated, when comparing with the control group (Figure 1b).

**Figure 2:**
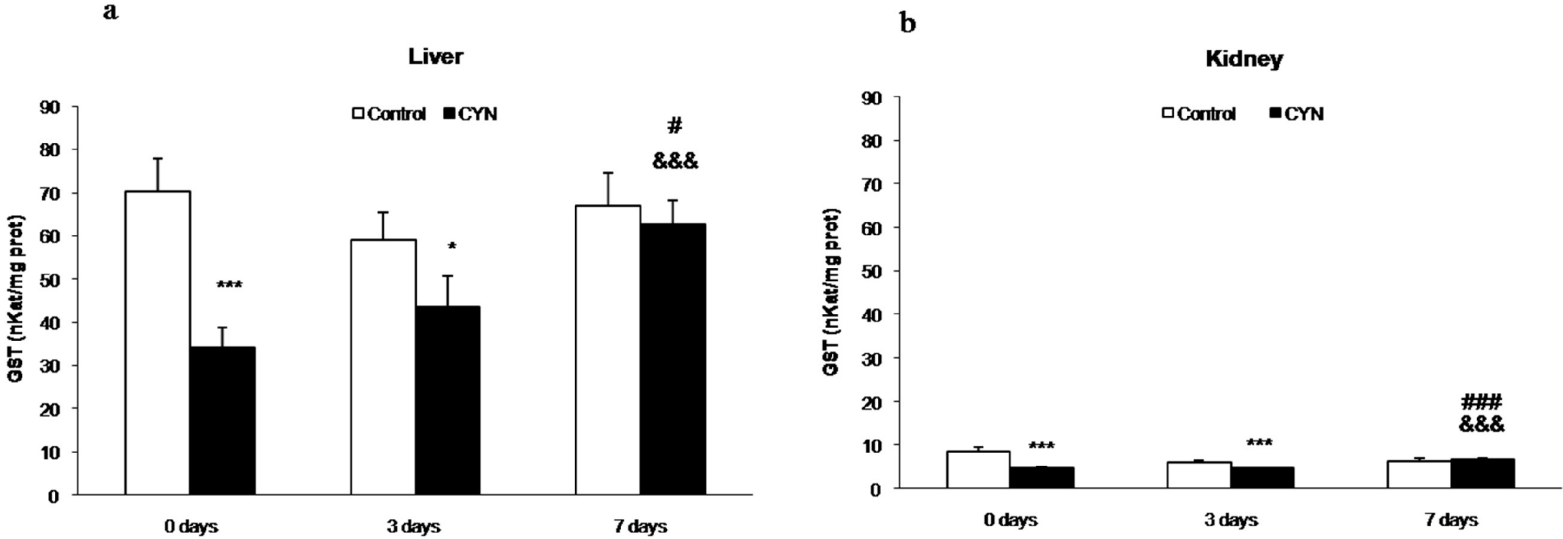
Glutathione-*S*-transferase (GST) activity (nkatals/mg protein) in liver (**a**) and kidney (**b**) of fish exposed by immersion to repeated doses of CYN-containing culture (equivalent to 10 µg CYN/L, added into aquaria every two days) for 14 days and later sacrificed (0 day) or depurated (3 or 7 days). The values are expressed as mean ± SE (*n* = 8). The significance levels observed are *** *p* < 0.001, * *p* < 0.05 in comparison with their respective control group, &&& *p* < 0.001 when CYN exposed and no depurated fish (0 day) and fish depurated at different times (3 or 7 days) are compared, and ### *p* < 0.001, # *p* < 0.05 when comparing CYN-depurated fish at 3 to 7 days of depuration.

sGST activities were decreased in both organs during the exposure time (0 day group, liver: 3.8-fold and 1.4-fold in kidney) and after the early days of depuration (3 days group, 1.8-fold and 1.3-fold in liver and kidney, respectively). After 7 days of depuration, the sGST levels in liver and kidney were restored to control values, and significant differences were detected in comparison to fish exposed to cyanobacterial cells containing CYN without depuration. Moreover, significant higher values were also observed after 7 days of depuration in comparison to 3 days of depuration (Figure 2a,b).

#### 2.1.2. Glutathione Peroxidase and Glutathione-*S*-Transferase Gene Expression

**Figure 3:**
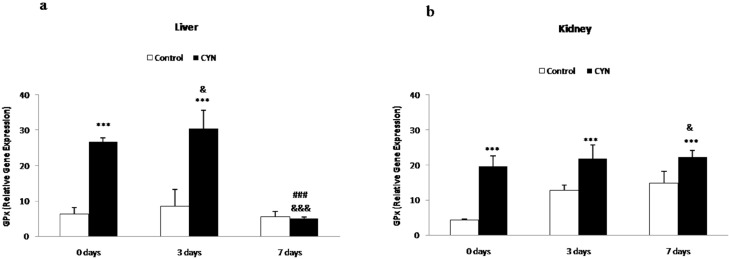
Relative gene expression of glutathione peroxidase (GPx) (×1000) in liver (**a**) and kidney (**b**) of fish exposed by immersion to repeated doses of CYN-containing culture (equivalent to 10 µg CYN/L, added into aquaria every two days) for 14 days and later sacrificed (0 day) or depurated (3 or 7 days). The values are expressed as mean ± SE (*n* = 8). The significance levels observed are *** *p* < 0.001 in comparison with their respective control group, &&& *p* < 0.001, & *p* < 0.05 when CYN exposed fish and then sacrificed and fish depurated at different times (3 or 7 days) are compared, and ### *p* < 0.001 when comparing CYN-depurated fish at 3 to 7days-depurated fish.

Significant alterations were observed in the relative gene expression of the enzyme GPx in the liver of fish exposed to cyanobacterial cells containing CYN non depurated and depurated for 3 days, in comparison to their respective control groups (4-fold and 3.5-fold up-regulation, respectively). No statistical differences were detected in fish of the longest depuration period (7 days) in comparison with their control group, and significant differences were evident between this group (7 days) and non-depurated fish and those subjected to 3 days of depuration (Figure 3a). In the kidney, all groups of fish showed significant increases in comparison to their respective control groups (4.5, 1.7 and 1.5- fold up-regulation, respectively) (Figure 3b).

**Figure 4:**
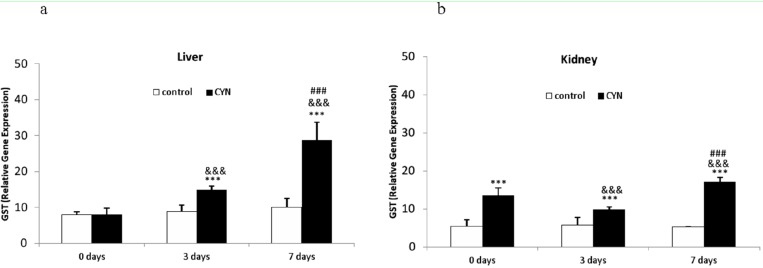
Relative gene expression of Glutathione-*S*-transferase (GST) (×1000) in liver (**a**) and kidney (**b**) of fish exposed by immersion to repeated doses of CYN-containing culture (equivalent to 10 µg CYN/L, added into aquaria every two days) for 14 d and later sacrificed (0 day) or depurated (3 or 7 days). The values are expressed as mean ± SE (*n* = 8). The significance levels observed are *** *p* < 0.001 in comparison with their respective control group, &&& *p* < 0.001 when CYN exposed fish and then sacrificed and fish depurated at different times (3 or 7 days) are compared, and ### *p* < 0.001 when comparing CYN-depurated fish at 3 to 7 days-depurated fish.

Regarding to sGST, both depuration processes assayed induced significant increases in the livers of fish in comparison to their respective control groups (1.7-fold and 2.8-fold for 3 or 7 days, respectively). Moreover, statistical differences were observed between fish exposed to CYN-containing culture and not depurated and those subjected to both depuration periods (1.8 and 3.5-fold for 3 or 7 days, respectively), which were more evident as time of depuration increased (7 days *vs.* 3 days) (Figure 4a). In the kidney, a significant enhancement was observed in all treated fish in comparison with their control groups (2.5-fold, 1.7-fold and 3-fold, respectively). Similar to the liver, significant differences were observed between both groups of CYN-exposed fish and those subjected to different depuration periods (7 days *vs.* 3 days) (1.7-fold) (Figure 4b).

#### 2.1.3. Glutathione-*S*-Transferase Protein Expression (Western Blotting)

**Figure 5:**
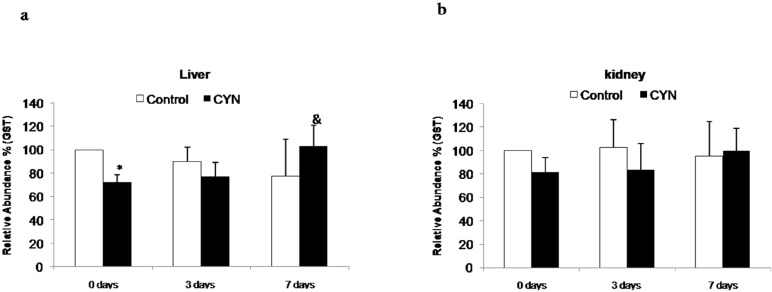
Relative abundance of Glutathione-S-transferase (GST) protein in liver (**a**) and kidney (**b**) of fish exposed by immersion to repeated doses of CYN-containing culture (equivalent to 10 µg CYN/L, added into aquaria every two days) for 14 days and later sacrificed (0 day) or depurated (3 or 7 days). The values are expressed as mean ± SE (*n* = 8). Results are expressed as relative abundance %. The significance levels observed are * *p* < 0.05 in comparison with their respective control group, & *p* < 0.05 when CYN exposed fish non depurated and fish depurated at different times (3 or 7 days) are compared.

The liver experienced a significant decrease in the protein expression of sGST in fish exposed to CYN and not depurated in comparison to their control group (0 day group). After depuration in clean water (3 or 7 days), no alterations in sGST expression were observed (Figure 5a). In the kidney, no significant changes were observed in any group of fish (Figure 5b).

#### 2.1.4. Determination of CYN in Water Samples from Aquaria

The initial concentration of CYN (after adding the first dose of CYN-containing culture into the aquaria) was 11.2 ± 0.5 µg/L and increased up to 42.4 ± 0.2 µg/L after 14 days of exposure. Once fish were removed from the aquaria, no traces of CYN were detected in the clean water during the depuration periods.

### 2.2. Discussion

Previous studies have reported that oxidative stress might play a role in the pathogenicity of CYN on tilapia exposed to a single dose [13,21] or repeated doses [23] of this molecule. However, the influence of different depuration periods on the changes in oxidative stress biomarkers induced by CYN has not been analyzed up to date.

The biochemical function of GPx is to reduce lipid hydroperoxides to their corresponding alcohols and to reduce free hydrogen peroxide to water. Fourteen days of exposure to CYN-containing culture of *A. ovalisporum* by immersion have resulted in changes of GPx activity only in the kidney. An increased susceptibility of this parameter to both pure CYN and CYN-containing cyanobacterial cells in the kidney of fish in comparison to the liver has also been found by other authors [13,23]. This could be related to the hydrophilic properties of CYN. Humpage and Falconer [36] considered that the kidney appeared to be the most sensitive organ in mice as well. Moreover, studies of the body distribution of 14C-labeled CYN in mice have shown that the main excretory route is through the kidneys, with nearly 50% of an intraperitoneally (i.p.) administered dose appearing in the urine within 6 h, and 20% of the dose present in the liver [37].

The increase observed both in the GPx activity and genetic expression is in accordance with the higher levels of lipid peroxidation products induced in these fish [38] and may indicate a defensive response of the fish to the toxic insult. Moreover, the increase observed in GPx activity is also related to the reduced values of GSH/GSSG observed in this organ [38] as GSH is being used in the catalytic activity of this enzyme to scavenge reactive oxygen species. GPx gene expression was significantly altered in both organs after 14 days of exposure to CYN-containing culture. This can be interpreted as a higher sensitivity of molecular biomarkers. Other authors have also found an absence of changes on GPx activity in the liver but on the contrary significant changes on *GPx* gene expression, although CYN concentrations and times of exposure were different [13,21]. 

Regarding to the effects of the depuration periods considered, no influence was found for GPx activity in the liver as CYN-containing culture did not cause any alteration in this organ after the exposure period. Similarly, Galanti *et al.* [33] did not observed changes in GPx activity neither in the accumulation period (3 days) nor in the detoxification period (6 days) in the shrimp *Palaemonetes argentinus* exposed to MC-LR under laboratory conditions. Ferreira *et al.* [26] found a significant decrease of GPx activity in the liver of mullets after one month of depuration from Douro estuary contaminants. Interestingly, after 4 months the activity increased and the authors attributed this finding to the higher temperatures of this period. In the kidney, the toxic response observed in fish exposed to cyanobacterial cells containing-CYN was recovered already after 3 days of depuration. This is in agreement with the reduction of LPO induction in these fish and the early recovery (only 3 days) of the GSH/oxidized glutathione (GSSG) levels [38] during the depuration process.

*GPx* gene expression in the liver required 7 days of depuration to decrease the enhancement induced by CYN-containing culture and to restore the basal values. However, in the kidney, 7 days were not enough, suggesting again a higher susceptibility of this organ in comparison to the liver. No other reports have been found in the scientific literature dealing with the influence of depuration periods on the genetic expression of oxidative stress biomarkers for this toxin.

GPx activity and gene expression showed the same pattern in the liver of intoxicated fish, with an increase from day 0 to day 3 and a decrease from day 3 to 7. However, there was no coincidence in other cases (exposure and depuration). This could suggest a post-transcriptional regulation of the enzyme or the combined effect of different *GPx* genes, as various forms of GPx are found in vertebrates [39,40].

GST is a well-known enzyme of Phase II of the metabolism of detoxication which conjugates glutathione to certain xenobiotics compounds or to their metabolites [41]. Nevertheless it has been demonstrated that GST shows peroxidase activities and may act in the antioxidative defence [42]. In this case, GST activity showed a significant decrease both in the liver and kidney. This finding is in agreement with the reduction of GSH/GSSG observed in these fish [38]. It is not known if GST is involved in CYN metabolism. Actually, the reported CYN metabolic route is a hepatic mixed function oxidation (CYP450) resulting in still unknown metabolites with a prominent role in CYN toxicity [43]. Considering this, the changes observed in GST activity could be attributed to its antioxidative role, as CYN-containing cultures have been demonstrated to increase the oxidation of lipids, DNA and proteins [38]. Previously, we had found a different response, namely an increase of the GST activity, but fish were exposed to pure CYN by acute gavage and intraperitoneal injection [13,21]. Changes of the response of this parameter can be observed even in the same experiment. Thus, Galanti *et al.* [33] reported an increase in the activity of the membrane bound GST exposed to MC-LR, whereas no change was observed in the soluble GST. Moreover, additional studies reported different *sGST* and *mGST* responses after MC-LR exposure in other organisms [44,45].

*sGST* gene expression showed a different pattern in comparison to the activity, with no changes in the liver and an increase in the kidney of fish exposed to CYN-containing cells for 14 days. Puerto *et al.* [13] also found the same response in the kidney but not in the liver. Again, different CYN concentrations and exposure time lead to different results. Moreover, responses of liver and kidney can be different, because the transcription of GST isoforms varies in different ways within an organ and among organs, as Li *et al.* [46] deduced from a study performed on goldfish exposed to MC-LR. There are different isoforms of GSTs, as they are a multiple gene family of dimeric enzymes, which can explain the differences observed between the enzymatic activity and the gene expression or even the differential response of the organs. Also, the absence of changes in *GST* expression after the intoxication period does not imply the same response after 14 days. He *et al.* [47] observed an increase of *GST* expression in tilapia exposed to MC-LR after 8 h of intoxication and, unexpectedly, a decrease after 24 h. 

In the case of sGST activity, the depuration process increased the values and allowed the restoration of the basal levels both in the liver and kidney in a time-dependent manner. This finding could be linked to the restoration of GSH/GSSG levels during the depuration process reported by Guzmán-Guillén *et al.* [38], as the activity of this enzyme depends on a steady supply of GSH. A similar response was observed by Özcan Oruç [27] in tilapia, in which case an increase of the GST activity was observed after 15 days of depuration following chlorpyrifos exposure. In *P. argentinus* Galanti *et al.* [33] also observed a significant increase of sGST and mGST during the detoxification period. Kankaanpää *et al.* [34] evaluated the influence of a 144 h depuration period in *Mytilus edulis* exposed for 24 h to nodularin, but they did not observe any change in the exposure period or in the depuration time*. sGST* gene expression of fish on the contrary, was not influenced by the depuration process in the kidney and resulted in higher values in the liver. This could be explained because between the gene expression of a particular enzyme and its final activity there are several molecular processes that can contribute to the differences observed.

sGST protein abundance decreased in the liver and did not show changes in the kidney. The reduction observed in the liver is in accordance with a well-known toxic mechanism of CYN, the inhibition of protein synthesis [48,49]. Thus, Humpage *et al.* [50] reported that the CYN parent compound was responsible for the inhibition of protein synthesis. This result is not in agreement with previous studies performed by Puerto *et al.* [13] and Gutiérrez-Praena *et al.* [21]. However, in those studies only a single dose of pure CYN was used and the observation period was shorter. The authors suggested that the dose employed was not enough or that the effect might need more time to be observed *in vivo*. In this regard, Kinnear [7] reported that CYN had a delayed toxicity involving multiple organ systems, principally the liver and kidneys. In this case, the continued exposure to CYN-containing cultures (equivalent to 10 µg CYN/L) for 14 days was enough to induce this toxic effect.

The relative abundance of sGST proteins did not correlate in all cases with the activity and the gene expression of the enzyme, suggesting that there is a regulation at the transcriptional and translational level or posttranslational modifications.

The depuration period for this parameter showed a positive influence on the liver, as values were restored to the basal levels already after 3 days. The effect on the kidneys could not be observed due to the fact that cyanobacterial cells that had been exposed to CYN did not affect this biomarker. 

The overall positive effects of the depuration periods on the toxicity induced by CYN on oxidative stress biomarkers have been reported previously for other contaminants [28,33,34,51]. These reports suggest that the time necessary to detect the influence of the depuration process is different for every contaminant, and it depends among other factors on the intensity of the toxic exposure, the detoxifying mechanisms available, the elimination kinetic of the toxic substance and the biomarker investigated.

## 3. Experimental Section

### 3.1. Chemicals and Reagents

CYN standard (purity > 95%) was supplied by Alexis Corporation (Lausen, Switzerland). Standard solutions of CYN were prepared in water milli Q (100 μg/mL, Millipore, Bedford, MA, USA) and diluted as required for their use as working solutions (0.08–5.0 μg/mL). All chemicals and reagents used for the different assays and analysis were purchased from Sigma-Aldrich (Madrid, Spain) and VWR International Eurolab S.L. (Seville, Spain). Deionized water (>18 MΩ cm^−1^ resistivity) was obtained from a Milli-Q water purification system (Millipore, Bedford, MA, USA). BOND ELUT^®^ Carbon cartridges (500 mg, 6 mL) were supplied by Agilent Technologies (Amstelveen, The Netherlands). Protein assay reagent was obtained from BioRad Laboratories (Hercules, CA, USA).

### 3.2. Aphanizomenon Ovalisporum Culture

*Aphanizomenon ovalisporum* (LEGE X-001) cyanobacterial CYN-producing strain (CYN+) was isolated from Lake Kinneret, Israel [52] and supplied by the Marine Research Center (Porto, Portugal). This strain was culture in Z8 medium, temperature of 25 °C, 28 µmol photons m^−2^s^−1^ of light intensity in continuous illumination. After 33 days, cultures were harvested by decanting with plankton net (20 µm diameter). The biomass obtained was frozen at –80 °C.

### 3.3. CYN Extraction and Analysis

For this study LEGE X-001 extract was analyzed by liquid chromatography-mass spectrometry (LC-MS)/MS in order to calculate the CYN present in the extract and produced per cell. CYN extraction from the culture of *A. ovalisporum* (CYN+) and determination by LC-MS/MS was performed based on Guzmán-Guillén *et al.* [53]. CYN was detected (retention time of 9.55 min) and quantified, and the concentration of CYN obtained was 0.1 μg CYN/mg of culture. Moreover, its deoxy-derivative (deoxy-CYN) has been also detected in the sample culture (retention time of 9.77 min), it was quantified using the CYN calibration curve (equivalent of CYN), and the concentration obtained was 0.012 μg deoxy-CYN/mg.

### 3.4. Experimental Set up and Fish Acclimation

Fish (Male *O. niloticus,* Nile tilapia, Perciformes: *Cichlidae*) were obtained from a fish hatchery “Aquaculture Valencia” and maintained at the laboratory to reach the average weight of 20 ± 8 g and 7.8 ± 2 cm length. Fish were held in aquaria (eight individuals/aquarium) with 96 L of fresh water. Exposure to chlorine was minimized by filling the tanks at least 3 days before the fish were introduced. The aquaria were also set up with a continuous system of water filtration and aeration (Eheim Liberty 150 (Eheim, Deizisau, Germany) and Bio-Espumador cartridges (Eheim, Deizisau, Germany)). The temperature was kept constant (21 ± 2 °C). Dissolved oxygen values were maintained between 6.5 and 7.5 mg/L. Mean values for additional parameters of water quality were: pH 7.6 ± 0.3, conductivity 287 µS/cm, Ca^2+^ 0.60 mM/L and Mg^2+^ 0.3 mM/L. Fish were fed with commercial fish food (ciprinidos, 2 mm, Dibaq, Segovia, Spain) and were acclimatized for 15 days before the beginning of the experiment.

### 3.5. Experimental Exposure

After the acclimation period, six groups of tilapia (*n* = 8) were established as is shown in Table 1.

**Table 1:** Treatment conditions of Tilapia fish (*Oreochromis niloticus*) exposed to *A.ovalisporum* (CYN+) culture by immersion.

Groups of Tilapia fish							
Treatment condition	Period of treatment	1	2	3	4	5	6
Exposure to *A. ovalisporum* (CYN+) culture	14 days	-	+	-	+	-	+
Depuration period	0 day	+	+	-	-	-	-
	3 days	-	-	+	+	-	-
	7 days	-	-	-	-	+	+

CYN+ = Cells containing Cylindrospermopsin; +, submitted to the treatment condition (exposed/depurated); -, not submitted to the treatment condition (exposed/depurated).

The experiment included a 14-days exposure period followed either by no depuration (Group 2), 3 days (Group 4) or 7 days depuration (Group 6). Controls were included for each experimental group for the same time period (Groups 1, 3, and 5). In addition, fish from Groups 2, 4, and 6 were exposed to CYN-containing culture of LEGE X-001 (Marine Research Center, Porto, Portugal) by immersion during 14 days. Cyanobacterial cells were added into the aquaria in an adequate quantity in order to obtain 10 µg CYN/L water. Afterwards, every 2 days the same amount of cyanobacterial cells was added to the aquaria during the exposure period. This concentration was selected in accordance with our previous experiment carried out in this fish species, in which 10 and 100 µg/L induced damage when fish were exposed sub-chronically by immersion with these concentrations [23]. All groups of fish were fed with commercial fish food (0.3 g/day). At the end of the exposure period, the fish from Aquaria 1–2 were sampled and sacrificed immediately. Fish in Aquarium 1 did not receive the toxin and were used as controls of 14 days of exposure to CYN. At the end of both depuration periods (3 or 7 days) fish were collected and sacrificed. Tilapias from Groups 3 and 5 were used as controls in comparison with fish in Aquaria 4 and 6 previously exposed for 14 days to CYN and subsequently depurated for 3 or 7 days, respectively. The procedure was designed to simulate the fish diet during and after a toxic *A. ovalisporum* bloom, whereas other feeding sources (represented by the fish food) were available at all times as well.

CYN total levels in the aquaria throughout exposure and depuration periods were measured every 48 h based on Guzmán-Guillén *et al.* [54] using LC-MS/MS, Briefly, 200 mL of water from each aquarium were subjected to freeze-thaw cycles twice as a cell disruption method, in combination with ultrasonication (15 min), and stirring (15 min). The resulting mixture was centrifuged at 4500 rpm for 15 min and filtered through a glass fiber filter (45 mm). For the clean-up procedure, graphitized carbon cartridges (Bond Elut^®^, Amstelveen, The Netherlands) were activated with 10 mL of a solvent mixture of dichloromethane (DCM) and methanol (MeOH) (10/90) acidified with 5% formic acid (*v*/*v*) and rinsed with 10 mL of MilliQ water. Subsequently, the sample was passed through the cartridges, washed with 10 mL of MilliQ water and eluted with 10 mL DCM/MeOH (10/90). In order to concentrate the sample, the extract was evaporated in a rotary evaporator and resuspended in 250 µL MilliQ water prior to its LC-MS/MS analysis. 

The limits of detection (LOD) and quantification (LOQ) of the toxin were 0.5 and 0.9 µg CYN/L, respectively.

### 3.6. Preparation of Postmitochondrial Supernatant (PMS)

Fish were anesthetized in ice water and sacrificed by transection of the spinal cord. The livers and kidneys were quickly removed, weighed, rinsed with ice-cold 0.9% saline solution, frozen in liquid nitrogen, and kept at –85 °C until use. Enzyme extracts from each tissue were prepared from each individual (not pooled) according to the method described by Puerto *et al.* [55]. Briefly, tissues were homogenized using 0.1 M potassium phosphate buffer (pH 6.5) containing 20% (*v*/*v*) glycerol, 1 mM ethylenediaminetetraacetic acid, and 1.4 mM dithioerythritol. After removal of cell debris (10 min at 13,000 *g*), the membrane fraction was separated by centrifugation at 105,000 *g* for 60 min. The remaining supernatant, defined as the soluble (cytosolic) fraction, was used for biochemical measurements.

### 3.7. Protein Estimation

Protein contents in the samples were estimated by the method of Bradford [56] using bovine γ-globulin as standard. Briefly, 5 μL of the diluted samples were mixed with 250 µL Coomassie Brilliant blue dye (Biorad Laboratories, Hercules, CA, USA) and the absorbance was read at 595 nm in the spectrophotometer (Tecan Infinite M200, Männedorf, Switzerland).

### 3.8. Glutathione Peroxidase and Glutathione-S-Transferase Activities

Glutathione peroxidase (GPx; EC 1.11.1.9) activity was assayed following the rate of NADPH oxidation at 340 nm by the coupled reaction with glutathione reductase [13]. The specific activity was determined using the extinction coefficient of 6.22 mM^−1^ cm^−1^.

Glutathione-*S*-transferase activity (sGST; EC 2.5.1.18) was measured in the liver and renal homogenates according to the method described by Habig *et al.* [57], by monitoring the formation of a conjugate at 340 nm and the formation of a conjugate between 16 mM GSH and 16 mM 1-chloro-2,4-dinitrobenzene (CDNB) [13]. The results of both enzymatic activities were expressed as nkat/mg protein.

### 3.9. RNA Preparation and Reverse Transcription

Total RNA was extracted and purified using the RNeasy Mini Kit^™^ (Cat # 74104, Qiagen, Madrid, Spain) according to the manufacturer’s instructions, as previously described by Puerto *et al.* [58]. The RNA integrity was assessed by agarose gel electrophoresis. RNA quality was assessed as the 260/280 nm absorbance ratio using a NanoDrop 2000 (Thermo Scientific, Pittsburgh, PA, USA). The RNA was then stored at −80 °C before further processing. Reverse transcription (RT) into cDNA was carried out with the High-Capacity cDNA Reverse Transcription Kit (Applied Biosystems, Madrid, Spain) following the manufacturer’s instructions. The cDNA was stored at −20 °C until further use.

### 3.10. Real-Time Polymerase Chain Reaction (RT-PCR)

A semi quantitative real-time polymares chain reaction (RT-PCR) protocol was developed to measure the mRNA levels of GPx, sGST in the liver and kidneys of tilapia, using beta-actin as protein control. The cDNA obtained was diluted in miliQ sterile water and used for the amplification by PCR. The forward and reverse primers used in this study are shown in Table 2.

**Table 2:** Nucleotides sequences (5'-3') of polymerase chain reaction (PCR) primers of beta-actine, GPx and sGST.

Gene	Forward primer	Reverse primer
*Beta-actine*	CAATGAGAGGTTCCGTTGC	AGGATTCCATACCAAGGAAGG
*GPx*	CCAAGAGAACTGCAAGAACGA	CAGGACACGTCATTCCTACAC
*GST*	TAATGGGAGAGGGAAGATGG	CTCTGCGATGTAATTCAGGA

All mRNA sequences were obtained from GenBank (DQ355022, EU234530, EF206801, for GPx, sGST and beta-actin, respectively). PCR primers for *GPx, sGST* and beta-actine were obtained from Sigma-Aldrich (Madrid, Spain). Each specific gene product was amplified by RT-PCR using LightCycler^®^480 System (Roche, Berlin, Germany) according to the following parameters: 50 °C for 2 min, 95 °C for 10 min, 95 °C for 15 s (40 cycles), 60 °C for 1 min (40 cycles), 72 °C for 10 min (40 cycles) and 4 °C until the pickup of the samples. Amplification data were collected by the sequence detector and analyzed with sequence detection software supplied by the manufacturer. For each assay, a standard curve was constructed using increasing amounts of cDNA. In all cases, the slope of the curves indicated adequate PCR conditions (slopes of 3.3–3.6). The RNA concentration in each sample was determined from the threshold cycle (*C*t) values and calculated with the sequence detection software supplied by the manufacturer (7000 SDS Software, Applied Biosystems, Pittsburgh, PA, USA) The quantitative fold changes in mRNA expression were determined relative to beta-actin mRNA levels in each corresponding group and calculated using the 2^−^^ΔΔ*C*T^ method × 1000 [59].

### 3.11. Western Blot Analysis for *sGST*

Liver and kidney tissues were prepared for measurement of sGST protein abundance by Western blot analysis. Homogenates (10% *w*/*v*) were prepared in 50 mM phosphate buffer, pH 7, and proteinase inhibitor (1%) at 0–4 °C with a polytron homogenizer RZR2102 (Heido, Germany). The homogenate was centrifuged at 2000 *g* for 10 min at 4 °C, and the supernatant was then used for the determination of total protein concentration [56]. Liver and kidney homogenized samples were mixed (1:1) with sample buffer containing β-mercaptoethanol (5%). Proteins were electrotransferred onto nitrocellulose membranes and immunoblotted as described by Mate *et al.* [60] using monoclonal anti-GST antibodies (Sigma-Adrich, Madrid, Spain), diluted at 1:3000 and 1:1000 in liver and kidney, respectively. The anti-GST antibody was detected by the enhanced chemiluminiscence (ECL) method according to the supplier’s protocol and using a peroxidase-conjugated antimouse IgG as a secondary antibody (1:2000 dilution). The image analysis software Multi Gauge 381472 was used (Fujifilm, Madrid, Spain). 

### 3.12. Statistical Analysis

All results were subjected to one-way analysis of variance (ANOVA), and represent means ± SE of eight fish per group (GraphPad Instat Sofware, La Jolla, CA, USA). Differences in mean values between groups were assessed by the Tukey’s test and were considered statistically different from *p* < 0.05.

## 4. Conclusions

In conclusion, the depuration process is an effective measure to mitigate the toxic effects induced by CYN-containing cyanobacterial cell exposure. GPx activity and sGST relative abundance recovered their basal values after only 3 days of depuration whereas GPx gene expression and sGST activity required 7 days. These results suggest that the oxidative stress effects produced in fish by cyanobacterial cells containing CYN could be restored after depuration processes and support the use of such types of damage as indicators of exposure to CYN-containing blooms.
